# Ultrasound-based deep learning radiomics nomogram for differentiating mass mastitis from invasive breast cancer

**DOI:** 10.1186/s12880-024-01353-x

**Published:** 2024-07-26

**Authors:** Linyong Wu, Songhua Li, Chaojun Wu, Shaofeng Wu, Yan Lin, Dayou Wei

**Affiliations:** Department of Medical Ultrasound, Maoming People’s Hospital, Maoming, 525011 Guangdong P. R. China

**Keywords:** Mass mastitis, Invasive breast cancer, Ultrasound, Deep learning radiomics, Differentiation

## Abstract

**Background:**

The purpose of this study is to develop and validate the potential value of the deep learning radiomics nomogram (DLRN) based on ultrasound to differentiate mass mastitis (MM) and invasive breast cancer (IBC).

**Methods:**

50 cases of MM and 180 cases of IBC with ultrasound Breast Imaging Reporting and Data System 4 category were recruited (training cohort, *n* = 161, validation cohort, *n* = 69). Based on PyRadiomics and ResNet50 extractors, radiomics and deep learning features were extracted, respectively. Based on supervised machine learning methods such as logistic regression, random forest, and support vector machine, as well as unsupervised machine learning methods using K-means clustering analysis, the differences in features between MM and IBC were analyzed to develop DLRN. The performance of DLRN had been evaluated by receiver operating characteristic curve, calibration, and clinical practicality.

**Results:**

Supervised machine learning results showed that compared with radiomics models, especially random forest models, deep learning models were better at recognizing MM and IBC. The area under the curve (AUC) of the validation cohort was 0.84, the accuracy was 0.83, the sensitivity was 0.73, and the specificity was 0.83. Compared to radiomics or deep learning models, DLRN even further improved discrimination ability (AUC of 0.90 and 0.90, accuracy of 0.83 and 0.88 for training and validation cohorts), which had better clinical benefits and good calibratability. In addition, the information heterogeneity of deep learning features in MM and IBC was validated again through unsupervised machine learning clustering analysis, indicating that MM had a unique features phenotype.

**Conclusion:**

The DLRN developed based on radiomics and deep learning features of ultrasound images has potential clinical value in effectively distinguishing between MM and IBC. DLRN breaks through visual limitations and quantifies more image information related to MM based on computers, further utilizing machine learning to effectively utilize this information for clinical decision-making. As DLRN becomes an autonomous screening system, it will improve the recognition rate of MM in grassroots hospitals and reduce the possibility of incorrect treatment and overtreatment.

## Introduction

Mass mastitis (MM) is a chronic, benign, non-specific inflammatory disease of the breast, which typically affects non-pregnant women aged 30 to 40 years old, and its incidence accounts for 1–5% of all breast diseases during the same period. The pathological features of MM mainly include suppurative mastitis, plasma cell mastitis and granulomatous mastitis [1]. Invasive breast cancer (IBC) is the most common breast mass type malignant disease, mainly affecting non-pregnant women above 40 years old, and accounting for 75–80% of all breast cancers during the same period. The pathological features of IBC mainly includes ductal and lobular carcinoma [2]. MM lacks specific clinical manifestations. In clinical practice, MM may present with breast masses, nipple invagination, ulcers, and axillary lymph node enlargement [3]. On ultrasound examination, MM can present as irregular hypoechoic masses, blurred edges, lobulated and other malignant signs [4]. MM may mimic IBC manifestations in clinical and imaging studies, especially those of invasive ductal carcinoma [5].

The clinical management of breast mass lesions is primarily through percutaneous needle biopsy or comprehensive visual imaging assessments, including ultrasound, mammography, and magnetic resonance imaging. However, due to the tumor heterogeneity, percutaneous needle biopsy results may not always be accurate, and invasive procedures can cause patient anxiety and discomfort. Visual imaging evaluations mainly rely on the American College of Radiology (ACR) Breast Imaging Reporting and Data System (BI-RADS) assessment, with some MM lesions classified into category 4 based on suspicious mammographic or sonographic findings [6]. These MM lesions often undergo surgical intervention with lumpectomy or mastectomy, and the management strategies for MM and IBC are different in clinical practice. MM often receives antimicrobial therapy, while surgical treatment is more common for IBC, which can have a significant psychological impact on patients [7]. Based on the above background, accurately identifying MM from BI-RADS 4 categories is of great significance for patients to avoid misdiagnosis and overtreatment.

However, due to the uneven distribution of medical imaging equipment and the differences in subjective diagnostic experience of ultrasound physicians, differentiating MM from IBC remains a formidable challenge [8]. Ultrasound, as the most widely used screening method for breast masses, also faces the aforementioned challenges. That is to say, visible imaging cannot meet the current demand for precise risk stratification in MM. In recent years, artificial intelligence (AI) has matured and developed in the field of medical imaging. AI is a technology based on computer technology that breaks through the visualization of medical images and quantifies the deeper information carried by medical images [9], including radiomics and deep learning. Radiomics and deep learning have been widely practiced in the diagnosis and treatment of breast cancer. For example, the ultrasound radiomics model developed based on 12 features could effectively distinguish triple negative breast cancer and fibroadenoma [10]; the deep learning network was trained and validated based on 1055 ultrasound images from two centers, and could predict the axillary lymph node metastasis in patients with primary breast cancer who were clinically negative [11]. More importantly, radiomics and deep learning have the possibility of complementing tumor information. As a new study method of combining two kinds of AI, deep learning radiomics (DLR) has become more and more fascinating in recent years. For example, ultrasound based deep learning radiomics nomogram (DLRN) was used to evaluate the pathological complete response of locally advanced breast cancer to neoadjuvant chemotherapy [12]. Interestingly, the value of DLRN in identifying MM and IBC has not yet been elucidated.

This study is the first to elucidate the application value of radiomics and deep learning features in identifying MM and IBC in ultrasound BI-RADS category 4 based on various machine learning algorithms, in order to accurately identify MM and reduce its risk classification for reducing the possibility of incorrect treatment and overtreatment. In addition, this study conducted a systematic review of the current study status on the identification of MM and IBC.

## Methods

### Breast study dataset

This retrospective study was conducted in accordance with the principles of the Helsinki Declaration and was approved by the Institutional Ethics Committee of Maoming People’s Hospital, which exempted patients from written informed consent. This breast cohort dataset was a review of all patients with breast mass lesions who underwent ultrasound examination at Maoming People’s Hospital from January 2020 to December 2022, and was based on the second edition of BI-RADS, each lesion was designated as category 4. Inclusion criteria: (1) Suspected breast lesions with mass, receiving ultrasound examination within 1 month; (2) Breast lesions were diagnosed as mastitis or invasive breast cancer by biopsy or surgery after ultrasound examination. Exclusion criteria: (1) Non pathologically confirmed breast lesions with mass; (2) Patients who receive any breast treatment before examination; (3) Ultrasound image loss or poor quality. Finally, 50 cases of MM and 180 cases of IBC were included, with an average age of 48 years, aged 21–86 years, including 87 cases BI-RADS 4a, 46 cases of BI-RADS 4b, and 97 cases of BI-RADS 4c.

### Ultrasound imaging protocol

**Fig. 1:**
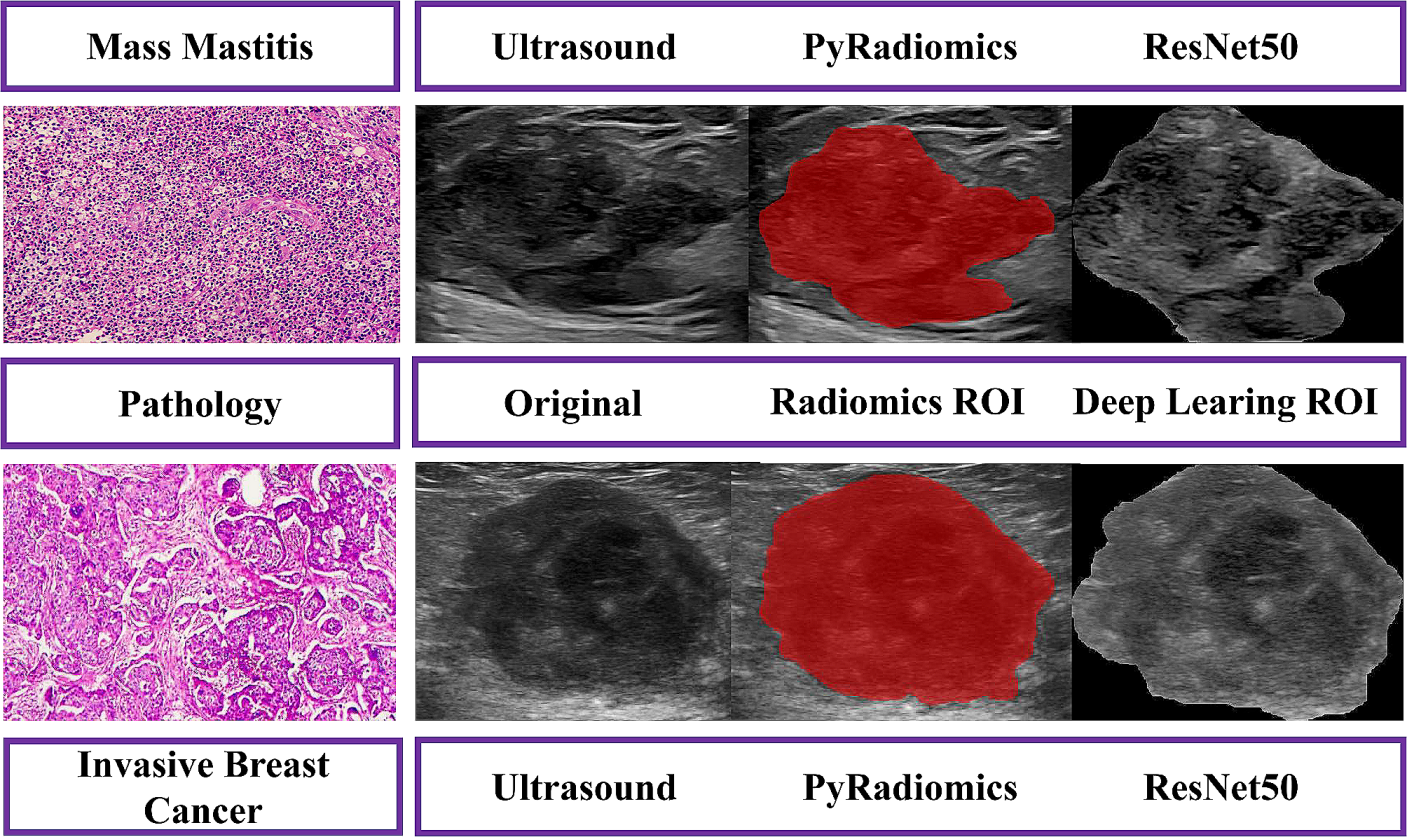
Representative achievements of MM and IBC

The acquisition of ultrasound images of breast mass lesions were based on various ultrasound diagnostic instruments: Mindray Resona7, LOGIQ E9/10, and Philips EPIC7C, equipped with high-frequency linear array probes (L11-3U; ML9L; and L12-5 and L12-3), with frequency of 5-13 MHz, and in a supine position (with both hands holding their heads). All ultrasound doctors had 3 years or more of experience in diagnosing breast lesions and were trained to collect images in standardized manners, which were the largest and clear images of the lesion. Image interpretation was based on BI-RADS, which was reviewed by two ultrasound doctors (one with over 10 years of work experience and the other with over 5 years of work experience). Subsequently, all breast lesions were analyzed in Medical Digital Imaging and Communication (DICOM) format and loaded into the ITK-SNAP 3.8.0 software [13]. The image edges interpretation (regions of interest, ROIs) were determined and described by the two ultrasound doctors mentioned above, and the clinical and histopathology data were unknown. Representative achievements of MM and IBC were shown in the Fig. 1.

### Radiomics and deep learning features extraction

All ROIs were resampled to a resolution of 1 mm × 1 mm × 1 mm. The ComBat method was applied to correct differences in radiomics feature changes resulting from variations in ultrasound equipment [14]. The Python programming software based on the PyRadiomics package was used to extract 1316 radiomics features from ultrasound images and transformed images for analysis of the imaging phenotype of breast mass lesions [15]. A total of 107 original features were extracted from the original image, including: shape features, first-order statistical features, gray-level co-occurrence matrix (GLCM) features, gray-level run length matrix (GLRLM) features, gray-level size zone matrix (GLSZM) features, gray-level dependence matrix (GLDM) features, and neighboring gray tone difference matrix (NGTDM). After image transformation, additional 1209 features were extracted: 744 wavelet features, 93 exponential features, 93 squareroot features, 93 gradient features, 93 logarithm features, and 93 square features. In addition, wavelet transform was an eight directional transformation of ROIs based on the order of high and low frequency components, obtaining eight types of wavelet features, namely HHH, HHL, HLH, LHH, LLL, LLH, LHL, and HLL.

The convolutional neural network model constructed a deep nonlinear neural network with multiple hidden layers, which was used to extract and combine low-level features layer by layer to form higher-level abstract features, avoiding the complex extraction process of traditional machine learning. The deep convolutional neural network model ResNet50 was used to extract deep learning features, consisting of 50 layers and was a shallow structured model [16]. The above ROIs were cropped to the maximum grayscale image of breast lesions based on the lesion boundary. The grayscale images had adopted random horizontal and vertical flipping enhancement strategies, randomly cropping to 224 × 224 pixels and outputting the images as the model input of the original images. The raw images and pathological labels of each breast lesion were input into the ResNet50 model. The ResNet50 model was pre trained using the ImageNet dataset, followed by transfer learning on the training cohort images [17]. During the training process, considering the output probability and pathological labels, the cross entropy loss function was used to update the network weights to complete the prediction, with a batch size of 64. After the training of the ResNet50 model was completed, the network parameters of the model were fixed, and then the fixed model was used as a feature extractor. A total of 2048 deep learning features were extracted from the second to last layer of the ResNet50 model in the training and validation cohorts.

### Deep learning radiomics nomogram development

To identify valuable differential features that were closely related to MM, features were analyzed using either the unpaired *t*-test if they were normally distributed or the *Mann-Whitney U* test otherwise. Features with a *P* value of less than 0.01 were screened out. Subsequently, based on the “caret” R packet, the breast cohort dataset was randomly divided into training cohort (*n* = 161) and validation cohort (*n* = 69) with a ratio of 7:3. In order to improve feature comparability, the features values of cohorts were normalized to the same order of magnitude using the Z-score method. In the training cohort, Pearson correlation coefficient was used for the correlation between features. If the correlation coefficient was greater than 0.99, one of the features were removed. The remaining features were presented in recursive feature estimation (RFE) [18], and the models were developed by selecting the optimal subset of features based on their importance. Logical regression (LR), random forest (RF) and support vector machine (SVM) machine learning algorithms were applied to develop radiomics and deep learning models based on the optimal feature subset, and optimize model parameters through five-fold cross validation to improve generalization and reduce error [19–21]. The validation cohort validated the generalization effect of the models based on the same feature subset and development parameters of the training cohort. In addition, radiomics and deep learning features had been validated to complement imaging phenotype information [22]. DLRN were developed based on the optimal subset of radiomics and deep learning features. The development process was the same as above.

### Deep learning phenotype to validate the heterogeneity of mass mastitis and invasive breast cancer

Machine learning can be divided into supervised learning and unsupervised learning. At present, most studies on unsupervised learning are based on the radiomics features, while the value of unsupervised learning based on the deep learning features have not been clarified. The above mentioned was the analysis of supervised learning. Unsupervised learning, that is, cluster analysis can achieve unbiased classification phenotype of anatomical structure by reducing the variability of observers, and can provide representative standard information for each classification phenotype [23]. K-mean clustering analysis is a mature unsupervised learning method, which was implemented based on the “ConsensusClusterPlus” package, and was used for deep learning feature phenotype classification in this study (parameter-setting: reps = 1000, pItem = 0.8, pFeature = 1, clusterAlg = km, distance = euclidean) [24]. The selected optimal deep learning feature subset was included.

### Literature retrieval based on ultrasound identification of MM and IBC

On the PubMed database, relevant literature was searched with the keyword “Breast AND Mastitis AND Ultrasound” to comprehensively review the literature on the differentiation of MM and IBC. Based on the full text of the literature, parameters were collected, including lead author, publication year, study cohort, and image type.

### Statistical analysis

SPSS 17.0 and R 3.68 software were used for statistical analysis and plotting. Continuous variables were represented by mean values ± standard deviations, and *t*-test was used for comparison; categorical variable were represented by examples (%), and Chi square test was used for comparison. The intraclass correlation efficient (ICC) was used to evaluate the stability of the optimal subset features among two different doctors. Univariate analysis was conducted to investigate the relationship between the optimal subset features and MM. The receiver operating characteristic curve (ROC) was applied to evaluate the performance of the models, and the area under the curve (AUC), accuracy (ACC), sensitivity (SEN), specificity (SPE) were calculated. Calibration curve analysis and Hosmer-Lemeshow (HL) test were used to evaluate models calibration and goodness of fit (*P* > 0.05 indicated that the models were well fitted). Delong test was used to evaluate the performance differences between different models [25]. Continuous Net Weight Classification Improvement (NRI) was used to evaluate the ability to improve classification effectiveness of models [26]. Decision Curve Analysis (DCA) was used to evaluate the net clinical benefit or utility of models [27]. *P* < 0.05 was considered statistically significant.

## Results

### Baseline analysis of breast cohort dataset

In this study, 50 cases of MM were included, including 27 cases of pure purulent mastitis, 5 cases of lymphoid plasma cell mastitis, 6 cases of granulomatous mastitis, and 12 cases of breast lobular purulent inflammation with granulomatous inflammation. There were 43 cases of BI-RADS 4a, 2 cases of BI-RADS 4b, and 5 cases of BI-RADS 4c; The average size of the lesions was 28.6 ± 13.7 mm, ranging from 8 to 71 mm. 180 cases of IBC were included, most of which were ductal carcinoma. There were 44 cases of BI-RADS 4a, 44 cases of BI-RADS 4b, and 92 cases of BI-RADS 4c; The average size of the lesions was 24.5 ± 10.6 mm, ranging from 7 to 64 mm. Age (*P* < 0.01), BI-RADS classification(*P* < 0.01), the difference between MM and IBC was statistically significant, and lesion size (*P* = 0.06) was not statistically significant. However, age (*P* = 0.46), lesion sizes (*P* = 0.05), and BI-RADS (*P* = 0.91) were not statistically significant in both the training and validation cohorts.

### Deep learning radiomics nomogram development

Through hypothesis test, 1316 radiomics and 2048 deep learning high-dimensional features were identified to be related to the nature of breast lesions with mass, leaving 281 radiomics features and 64 deep learning features (*P* < 0.01). The remaining features were further removed from 69 and 0, 195 and 45 radiomics features and deep learning features through PCC and RFE, respectively. Finally, machine learning models were developed based on 17 radiomics features and 19 deep learning features, which showed good consistency under the depiction experience of different ultrasound physicians, with ICC ≥ 0.75. Through univariate analysis, 9 radiomics features and 14 deep learning features were determined to be closely related to MM, which the odds ratio (OR) values of 8 features were ≥ 1.48, and 9 features were ≥ 1.45, respectively.

Table 1 showed the predictive performance of radiomics and deep learning models for MM. In the radiomics models, the RF models performed better than LR or SVM models, which prediction performance in the training and validation cohort was as follows: AUCs were 0.73 [95% confidence interval (CI), 0.64–0.82] and 0.76 (95% CI, 0.62–0.87), ACC were 0.64 and 0.67, SEN were 0.80 and 0.93, SPE were 0.60 and 0.59, respectively. Interestingly, the RF models performed equally well in deep learning models, which predictive performance in training and validation cohort was as follows: AUCs were 0.87 (95%CI, 0.80–0.93) and 0.84 (95%CI, 0.71–0.94), ACC were 0.78 and 0.81, SEN were 0.91 and 0.73, SPE were 0.74 and 0.83, respectively. The above analysis summarized that the deep learning feature appears to be superior to the radiomics feature in all machine learning, and in the validation cohort, AUCs ≥ 0.75, ACC ≥ 0.65, SEN ≥ 0.73, and SPE ≥ 0.61. Unfortunately, further evaluation through the Delong validation revealed that there was no significant performance difference between radiomics and deep learning models, except for the RF training cohort model (*P* = 0.01) and the SVM validation cohort model (*P* = 0.03).

**Table 1:** Performance of radiomics and deep learning machine learning models

		Training cohort							Validation cohort					
Models		AUC	ACC	SEN	SPE	PPV	NPV		AUC	ACC	SEN	SPE	PPV	NPV
SVM	RAD	0.75	0.81	0.46	0.91	0.59	0.86		0.64	0.61	0.67	0.59	0.31	0.86
RF		0.73	0.64	0.80	0.60	0.35	0.91		0.76	0.67	0.93	0.59	0.39	0.97
LR		0.81	0.81	0.77	0.82	0.54	0.93		0.60	0.51	0.80	0.43	0.28	0.88
SVM	DL	0.84	0.83	0.80	0.83	0.57	0.94		0.84	0.80	0.87	0.78	0.52	0.95
RF		0.87	0.78	0.91	0.74	0.49	0.97		0.84	0.81	0.73	0.83	0.55	0.92
LR		0.87	0.87	0.69	0.92	0.71	0.91		0.75	0.65	0.80	0.61	0.36	0.92

Note: SVM support vector machine; RF random forest; LR logical regression; RAD, radiomics; DL, deep learning; AUC, area under the curve; ACC, accuracy; SEN, sensitivity; SPE, specificity; PPV, positive predictive value; NPV, negative predictive value.

**Fig. 2:**
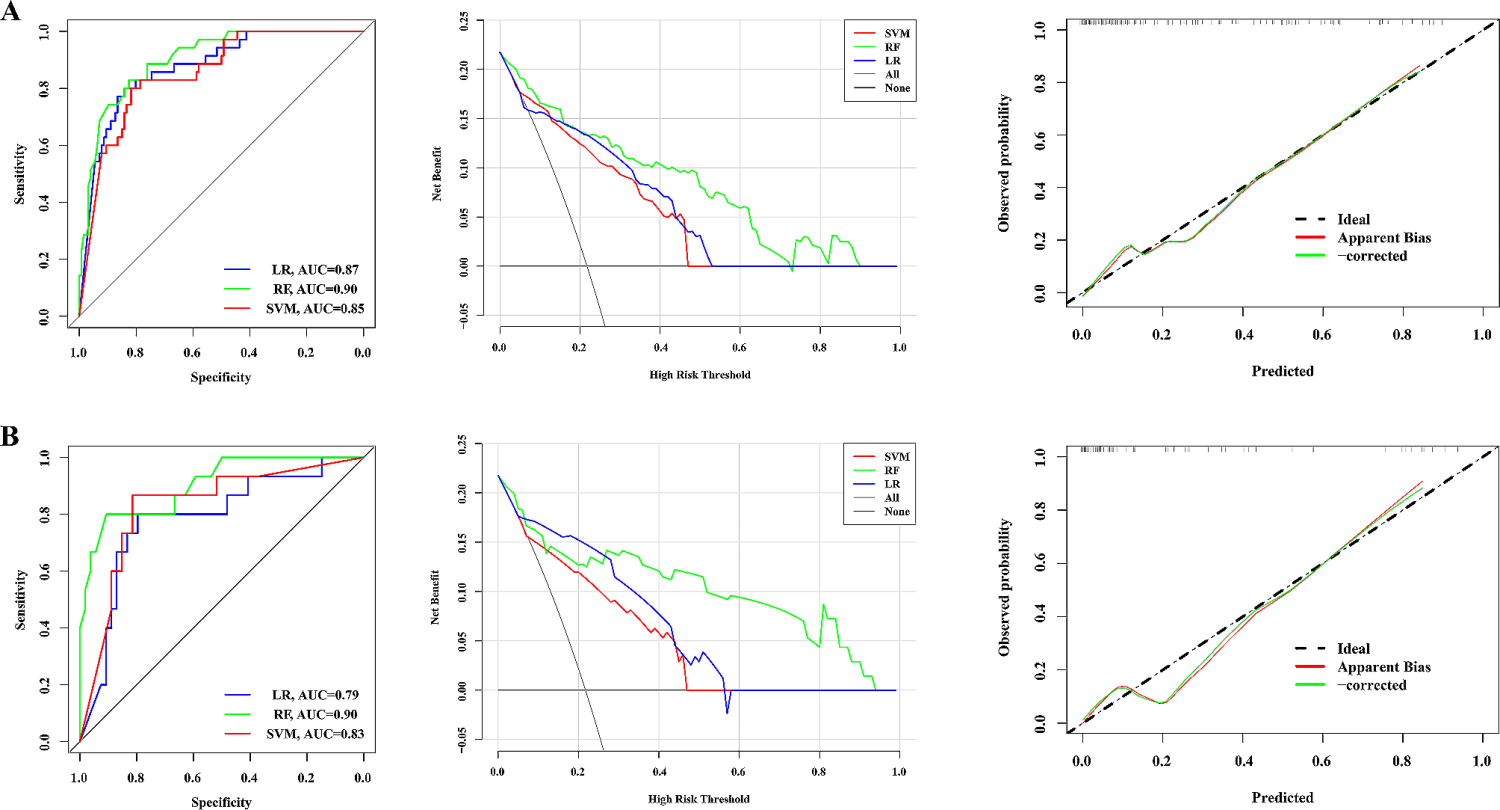
Performance of deep learning radiomics nomogram in the training cohort (**A**) and validation cohort (**B**). The RF-DLRN was shown to have better discriminatory performance, clinical benefit and calibrability

Based on 17 radiomics features and 19 deep learning features, DLRN were developed by integrating 5 radiomics and 10 deep learning features under the same process, and their predictive performance were shown in Table 2. The RF model still performed well, which predictive performance in training and validation cohort was as follows: AUCs were 0.90 (95%CI, 0.85–0.96) and 0.90 (95%CI, 0.80–0.98), ACC were 0.83 and 0.88, SEN were 0.83 and 0.80, SPE were 0.83 and 0.80, respectively. Unfortunately, the Delong test found no performance difference between the DLRN nomogram and deep learning models. However, compared to deep learning models, continuous NRI showed significant improvement in the identification and reclassification of MM in the remaining cohorts (*P* < 0.05), excluding SVM-DLRN in the training cohort. Specifically, continuous NRI showed RF-DLRN improved by 48% (*P* = 0.009) in the training cohort and 61% (*P* = 0.03) in the validation cohort. It was not difficult to see the superiority of the RF models in developing two types of features. Furthermore, the calibration curve and Hosmer-Lemeshow test showed that both the RF-DLRN training cohort (*P* = 0.91) and validation cohort (*P* = 0.22) had good consistency between prediction and pathological reality. DCA demonstrated the excellent performance of DLRN in clinical decision-making, which compared to single radiomics or deep learning model, DLRN had greater potential clinical application value and could provide better net benefits (Fig. 2).

**Table 2:** Performance of deep learning radiomics nomogram

	Training cohort							Validation cohort					
Nomogram	AUC	ACC	SEN	SPE	PPV	NPV		AUC	ACC	SEN	SPE	PPV	NPV
SVM	0.85	0.81	0.80	0.82	0.55	0.94		0.83	0.83	0.87	0.81	0.57	0.96
RF	0.90	0.83	0.83	0.83	0.57	0.95		0.90	0.88	0.80	0.91	0.71	0.94
LR	0.88	0.83	0.80	0.84	0.58	0.94		0.79	0.80	0.80	0.80	0.52	0.93

### Deep learning phenotype to validate the image heterogeneity of mass mastitis and invasive breast cancer

**Fig. 3:**
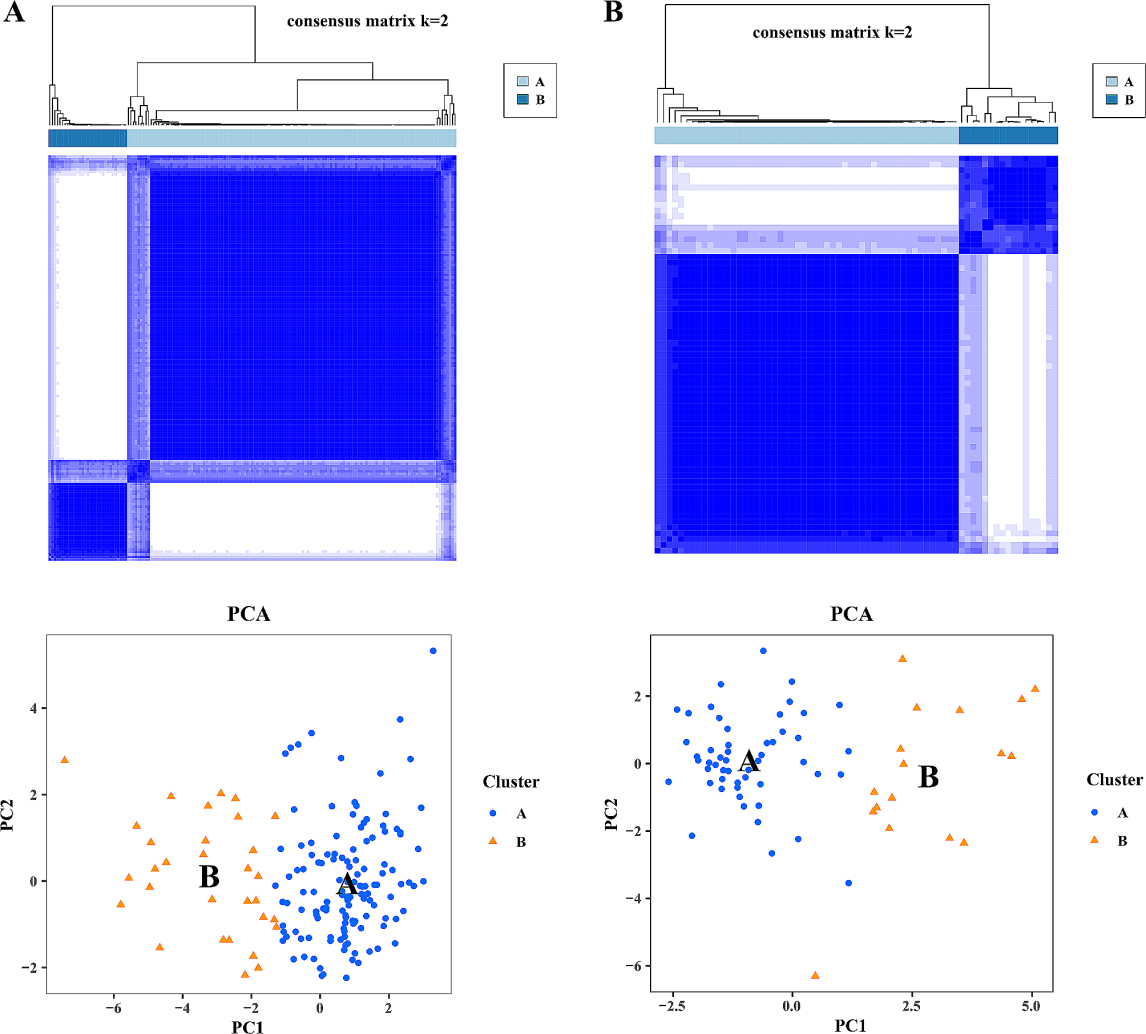
Deep learning phenotype to validate the image heterogeneity of mass mastitis and invasive breast cancer. K-means clustering analysis on 19 deep learning differential features, the clustering effect also was best when K = 2 in the training and validation cohorts, respectively. The distribution of MM and IBC in these two clusters was different (training cohort, *P* < 0.01; validation cohort, *P* < 0.01)

By conducting K-means clustering analysis on 19 deep learning differential features, the clustering effect also was best when K = 2 in the training and validation cohorts, respectively. That was to say, through deep learning features, 230 patients could be divided into two clusters (A and B) based on image heterogeneity. In addition, the results of principal component analysis also indicated that deep learning effectively divided patients into two clusters, validating the results of K-means clustering analysis. Among them, in the training and validation cohorts, there were 130 and 52 cases, 31 and 17 cases were divided into cluster A and cluster B. More excitedly, the distribution of MM and IBC in these two clusters was different (training cohort, *P* < 0.01; validation cohort, *P* < 0.01), MM was more concentrated in cluster B, and IBC was more concentrated in cluster A (Fig. 3). This confirmed and reaffirmed the difference of imaging phenotypes between MM and IBC, that was, MM had its own specific imaging information.

### Literature retrieval based on ultrasound identification of MM and IBC

Of the 628 literature, 12 were identified to be related to the differentiation between MM and IBC, of which 2 were artificial intelligence (AI) studies (Table 3). One study on AI was on radiomics, and the other as on deep learning. Among the other 10 studies, 2 were contrast-enhanced ultrasound studies, 1 was ultrasound combined with superb microvascular imaging studies, and 7 were ultrasound combined with elastic studies. Types of mastitis included plasma cell mastitis and granulomatous mastitis. Nine studies evaluated using AUC, sensitivity, and specificity; One study only used AUC for evaluation; Two studies were evaluated using differential statistical analysis [5, 28–38].

**Table 3:** Literature retrieval based on ultrasound identification of MM and IBC

Author	Year	Mastitis (*n*)	Malignant breast lesions (*n*)	Image type
Yağcı B et al.	2017	23	45	US + SWE
Teke M et al.	2017	48	122	US + SWE
Yao C et al.	2018	37	122	US + SWE
Liu SQ et al.	2018	26	79	US + SWE
Arslan S et al.	2018	36	77	US + SWE
Zhu YC et al.	2019	17	78	US + SMI
Makal GB et al.	2021	88	80	US + SWE
Toprak N et al.	2022	39	94	US + SWE
Zheng Y et al-1	2022	88	91	CEUS
Yin L et al.	2022	30	58	CEUS
Zhou Y et al.	2022	232	707	US (deep learing)
Zheng Y et al-2	2022	40	40	US + CEUS (radiomics)

Note: US, ultrasound; SWE, shear-wave elastography; SMI, superb microvascular imaging; CEUS, contrast-enhanced ultrasound

## Discussion

The purpose of this study was to develop and validate machine learning models developed based on radiomics features and deep learning features of ultrasound images that could identify MM from breast disease for potential clinical value. The supervised machine learning results showed that the deep learning models were better able to identify MM and IBC compared to the radiomics models, especially the RF model, with an AUC of 0.84, ACC of 0.83, SEN of 0.73, and SPE of 0.83 for the validation cohort. Moreover, compared to the radiomics or deep learning models, DLRN even further improved discrimination (AUC of 0.90 and 0.90, ACC of 0.83 and 0.88 for training and validation cohorts) with better clinical benefit and good calibrability. In addition, the information heterogeneity of deep learning features in MM and IBC was again validated by unsupervised machine learning clustering analysis, indicating that MM has unique imaging-informed phenotype and can potentially be applied to clinical practice to increase the recognition rate of MM.

Visual imaging still faces great clinical challenges in distinguishing the same imaging manifestations of different diseases. MM can mimic IBC by presenting with the same visual features, such as crab foot shape, burr shape. For inexperienced primary doctors, MM can easily be misdiagnosed as IBC to the point of classification as a high-risk group classified in BI-RADS category 4 or even higher. In the present study, 86% (43/50) of MM were classified as BI-RADS 4a, 51% (92/180) of IBC were classified as BI-RADS 4c, and still 24% (44/180) of IBC were classified as BI-RADS 4a, which is similar to the previous studies [35]. Clinical management of breast lesions with mass in BI-RADS category 4 is likely to be interventional and no longer an observational strategy [39]. However, there is a clear differential benefit in the treatment strategies for IBC and MM. Preoperative noninvasive imaging evaluation is particularly important, and conversely it is clear from the above results that conventional ultrasound was no longer well suited to such clinical needs. Therefore, in recent years, many studies have further expanded this part of the study based on conventional ultrasound. Previous studies were summarized in this study, and it was found that ultrasound combined with elastography or ultrasonography was two techniques that had been studied more often, especially elastography. For example, shear wave velocity was significantly different in 26 cases of mastitis and 79 cases of breast malignancy with an AUC of 0.70 [30]; quantitative parameters of ultrasonography allowed risk downgrading in 30 cases of MM with an AUC of 0.81 [36]. However, elastography is time-consuming and credibility assessment causes it to remain difficult to be widely available in the clinic; ultrasonography is not sufficiently performed in local hospitals. These are the clinical challenges that limit the realization of precision medicine.

Elastic imaging quantifies the hardness of tumors, while contrast-enhanced ultrasound quantifies the blood supply of tumors. A question arises whether image information can be quantified on a large scale using methods similar to gene sequencing. In recent years, the application of AI in medical imaging has impacted the field of visual imaging and is more likely to become the cornerstone of precision medicine [40]. AI, as a high-throughput information quantification technology that includes internal texture, edge, shape and other information, has been widely used in breast diseases. For example, radiomics machine learning models developed based on 5234 ultrasound image features could predict multiple molecular expressions of Ki67, p16, and p53 [41]; Based on the deep learning model developed from 937 ultrasound images of breast cancer patients, through testing, the SEN of identifying lymph node metastasis of breast cancer reached 0.98 [42]. Compared to elastic imaging and contrast-enhanced ultrasound, AI contains a greater amount of information, but it may also contain a lot of redundant information. The application of AI in MM and IBC had only found a limited number of studies, such as, automatic classification of 512 breast malignant nodules and 255 breast inflammatory nodules based on deep learning algorithms, which had a high coincidence rate with postoperative pathology and a low coincidence rate with inexperienced ultrasound physicians [37]. The radiomics features based on contrast-enhanced ultrasound and conventional ultrasound could effectively distinguished 129 breast inflammatory nodules and 125 breast nausea nodules, with AUC and accuracy estimated at 0.80 and 0.76 in the validation cohort, respectively [38]. However, DLRN study combining radiomics and deep learning features, as a recent study hotspot, is still lacking in the identification applications of MM and IBC.

Therefore, the present study filled a gap in the application of DLRN for MM and IBC identification. First, the quality was assessed based on the radiomics quality score method, and the quality score of this study was found to be 18/36, which was in the medium to high quality. Second, this study used supervised and unsupervised machine learning forms, and supervised learning was used to compare three classifiers: LR, RF and SVM. Since the features were in high dimension and there were redundant or worthless features, a combination of statistical tests, correlation coefficients, and RFE were used to reduce the dimensionality and the results showed good results. Seventeen radiomics and 19 deep learning high-value features were selected for dimensionality reduction and the risk of features was verified by one-way analysis. The RF classifier performed best in both the radiomics models and the deep learning models. However, Delong test showed that the RF-deep learning model outperformed the radiomics model only in the training cohort and was not statistically significant in the validation cohort. Further, DLRN was developed based on 5 radiomics and 10 deep learning features. Unfortunately, Delong test found no performance difference between the DLRN and deep learning models. The most likely explanation for the Delong test results were the insufficient sample size and the impact of human intervention during the derivation process of deep learning networks. In addition, the deep learning model exhibited better AUC values than the radiomics model, which may be affected by overfitting questions in deep learning model data processing [43]. However, continuous NRI showed a 48% improvement (*P* = 0.009) for RF-DLRN in the training cohort and a 61% improvement (*P* = 0.03) in the validation cohort. The multi-metric assessment used in this study is more rigorous compared to other studies’ performance comparisons. In addition, DLRN had better calibrability and clinical benefit through calibration curve and decision curve analysis. From the above, it was clear that supervised machine learning methods had potential value in MM and IBC identification. Finally, by unsupervised learning-K-mean clustering analysis, it was found that the deep learning features could classify patients into two clusters (A and B), with MM more concentrated in cluster A and IBC more concentrated in cluster B. The difference was statistically significant. That was, both MM and IBC had their own unique imaging phenotypes, validating the reliability of supervised learning.

This study also had limitations. Firstly, this study was based on single center data from grassroots hospitals, which lacked generalization ability and still required large sample multi center validation. Secondly, multiple ultrasound diagnostic instruments were used in this study, and differences in imaging protocols resulted in differences in the information carried by the images, which affected the credibility of the results. Third, PCC did not compare multiple threshold settings to determine the impact on model development. Finally, the potential value cannot be compared without comparison with transfer deep learning, mage fusion radiomics and pathomic [44, 45].

## Conclusions

In summary, DLRNs developed based on radiomics and deep learning features of ultrasound images have potential clinical value in effectively differentiating MM and IBC, which can be provided as an auxiliary system for reference by inexperienced junior doctors for reducing the possibility of incorrect treatment and overtreatment. In addition, this study used supervised and unsupervised machine learning forms to mutually validate the value of imaging phenotypes in differentiating MM and IBC, indicating that both have their own unique imaging phenotypes, with a view to future validation and translation of clinical results.

## Data Availability

The data sets generated and/or analyzed by the current study can be obtained from the corresponding author upon reasonable request.
